# Surface roughness and color measurements of glazed or polished hybrid, feldspathic, and Zirconia CAD/CAM restorative materials after hot and cold coffee immersion

**DOI:** 10.1186/s12903-021-01770-2

**Published:** 2021-08-30

**Authors:** Lujain I. Aldosari, Abdulkhaliq A. Alshadidi, Amit Porwal, Nasser M. Al Ahmari, Mohammed M. Al Moaleem, Ahmed M. Suhluli, Mansoor Shariff, Ahmed O. Shami

**Affiliations:** 1 Prosthetic Department, College of Dentistry, King Khalid University, Abha, Kingdom of Saudi Arabia; 2 Applied Medical Sciences College, King Khalid University, Abha, Kingdom of Saudi Arabia; 3 Department of Prosthetic Dental Science, College of Dentistry, Jazan University, Jazan, 45142 Kingdom of Saudi Arabia; 4 Intern Department, College of Dentistry, Jazan University, Jazan, Kingdom of Saudi Arabia

**Keywords:** Arabic Qahwa, Cold coffee, CAD/CAM, Surface roughness

## Abstract

**Background:**

The purpose of this study evaluates and compares the effect of surface roughness (Ra) and color stability on computer-aided design/computer-aided manufacturing (CAD/CAM) hybrid resin (Vita Enamic), feldspathic (Vitablocs® Mark II), and lithium disilicate Zirconia (Vita Suprinity) glazed or polished ceramics immersed in hot Arabic Qahwa and cold coffee.

**Methods:**

A total of 96 standardized samples were prepared from CAD/CAM restorative materials. Half of the samples were polished as per the manufacturer’s instructions using a porcelain polishing kit, and the other half were glazed. Samples were distributed and immersed in hot Arabian Qahwa and cold coffee followed by thermocycling. Ra measurements and color changes were conducted before and after immersion. SEM images were captured from each type of glazed or polished ceramic. One-way ANOVA paired Student’s t-test, and Bonferroni test were conducted to detect significant difference between the groups. P > 0.05 was a significant level.

**Results:**

Of all the tested samples, Ra increased without any significant difference; however, mean color changes (ΔE*) showed significant differences. An increase in Ra was noted for all the glazed and polished samples after immersion and thermocycling. However, differences were significant only in VM II. In addition, ΔE* was significant only in Vita Suprinity (VS) samples. For immersion groups, significant Ra changes were noticed in glazed samples, only in Vita Enamic (VE) with no ΔE*. In polished samples, mean Ra changes were observed in VM II and VS samples. Significant differences were also noticed in polished VE and VS subgroups of ΔE*.

**Conclusions:**

Ra affects all the tested samples, providing higher values on the polished specimens. The ΔE* caused by hot Arabic Qahwa and cold coffee on glazed or polished CAD/CAM restorative materials were clinically acceptable.

## Background

In the last few years, patients are increasingly demanding long-term color stability of aesthetic restorations to improve teeth appearance [1–3]. To meet the demand, computer-aided design/ computer-aided manufacturing (CAD/CAM) materials have been developed to fabricate the all-ceramic restoration materials. Over the last decades, the CAD/CAM systems have enhanced new all-ceramic materials for the restoration of the esthetic zone, attributed to the restoration of aesthetics or clinical longevity [4]. Dental hybrid-based ceramic is one of the most popular used material to fabricate all-ceramic prostheses in modern dental practice owing to the benefits in term of higher mechanical properties [5–7] and outstanding biocompatibility compared to other ceramic restorative materials [8–10].

The surface roughness (Ra) and the color stability are other successes of the restorations in the esthetic zone area. Ra values are used to assess Ra materials and compare their efficacy [11]. Alp et al. [12] showed that the surface treatments using the Vita Suprinity (VS) restorative materials are clinically acceptable for color changes after coffee staining and thermocycling. Egilmez et al. [13] stated that CAD/CAM Vita Enamic (VE**)** restorative materials exhibited different Ra values and surface topographies. After immersion in Khat extract, another study found lower Ra values for glazed or polished metal-ceramic as compared with glazed Vitablocs Mark II (VM II) [14]. Some studies concluded that low fusing or feldspathic porcelain had stable surfaces after immersion in different staining materials [15–18]. Al Moaleem et al. [14] showed a higher significant effect on Ra of CAD/CAM Zirconia compared to feldspathic ceramic, manually or machinable packed CAD/CAM materials.

An excellent aesthetic ceramic restoration is desired. In addition, maintaining the protheses' color stability is crucial in the oral environment. Kursoglu et al. [19] claimed that diet, immersion time, and categories of porcelain surface were responsible for prosthetic material discoloration. However, other studies evaluated the color porcelain materials stability concerning the surface texture, concluding that accurate polishing procedures could create smooth ceramic surfaces similar to glazed surface [20, 21]. Polishing techniques as an alternative to glazing have also been suggested [19–21].

The CIE Lab color system is global color research that interprets clinically values of colors and differences in color changes (ΔE*). Based on the color-magnitude between two objects, color spaces are comprised of three coordinates: L*(lightness, brightness, black/white color character), a*, and b* (chromatic color characters) [22–24].

Coffee causes discoloration to all-ceramic prostheses. Gupta et al. [20] evaluated the ceramic materials' color stability after exposure to commonly consumed beverages i.e., tea, coffee, and Coca-Cola. They concluded that dietary beverages affected the ceramic restorations. Alghazali et al. [22] discovered that Arabic coffee changed the color of different types of ceramic materials either glazed or polished forms. In addition, coffee immersion significantly changed Zirconia ceramic colors [12, 18, 25].

Coffee is one of the most popular consumed beverages worldwide. The Saudi population consumes a special type of coffee called ‘’Arabian Qahwa’’. It contains some additives: Saffron, Ginger, and Cardamom. However, Arabian Qahwa is a discoloration drink due to additive constituents, which affects teeth stain during aesthetic restorations [22, 26]. With an increase in coffee consumption in Saudi and globally, esthetic restorative procedures are crucial to conserve the maximum amount of original quality teeth. Recent studies evaluate materials for aesthetic restorations. However, few focused on the recent advanced CAD/CAM materials for aesthetic restorations of the Arabic Qahwa and cold coffee consumers.

This in-vitro study aimed to assess the effect of glazing or polishing on Ra and color stability of resin matrix glass–ceramic VE, feldspathic ceramic (Vitablocs® Mark II), and lithium disilicate Zirconia (Vita Suprinity) CAD/CAM restorative ceramic materials following exposure to staining solutions, such as Arabic Qahwa (hot) and cold coffee drinks along with thermocycling.

*Null hypothesis*: No mean color changes (ΔE*) or mean Ra changes in the CAD/CAM restorative materials groups and the individual group after surface treatment, immersion solutions, and thermocycling. In the current study, comparison of the results will be calculated based on 50: 50% perceptibility and acceptability thresholds (AT and PT).

## Methods

### Study design

**Fig. 1:**
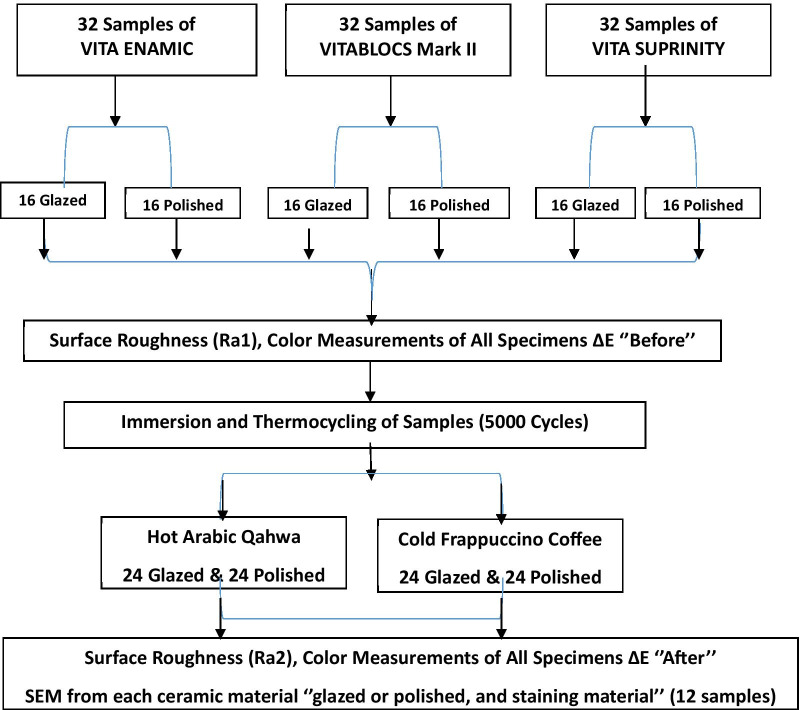
Study design and sample distribution of all CAD/CAM materials along with the surface treatments and measurements (Ra and ΔE and SEM)

The design included different glazed or polished CAD/CAM all-ceramic samples (96 overall) to simulate oral cavity, assess changes in the surface roughness and color after immersion (30 days) in hot Arabic Qahwa, cold (Frappuccino) coffee drink, and thermocycling. Table 1 presents the materials and devices. Figure 1 presents the flow chart of the study design, steps, and sample distributions.

**Table 1:** Materials and devices used in the study

Material/Device Type	Brand Name	Composition	Manufacturers	Lot #	Shades
Hybrid glass ceramic in resin matrix	VITA ENAMIC (VE)	Polymer infiltrated feldspathic ceramic network material with 86 wt% ceramic, UDMA, TEGDMA	VITA Zahnfabrik, Bad Säckingen, Germany	51960	1M2HT
Feldspathic glass ceramic	Vitablocs Mark II (VM II)	Fine-particle feldspar glass ceramic 30%	VITA Zahnfabrik, Bad Säckingen, Germany	42650	IM2C
Zirconia reinforced lithium disilicate glass ceramic	VITA SUPRINITY (VS)	silicon dioxide (56–64%), Li_2_o (15–21%), Zro_2_ (8–12%), La_2_O_3_ (0.1%), and pigments	VITA Zahnfabrik, Bad Säckingen, Germany	40880	1M2HT
Porcelain Polishing kit	OptraFine	Multi-step diamond finishing and polishing system for ceramics	Ivoclar, Vivadent, Clincal, Ag, FL Schann Liechtenstein	Lot XL0690, REF # 601989AN Expiry date—2021-09-04	NA
Surface Roughness Tester	Profilometer	Samples were measured by Vertical Scan Interferometry using 5 × Michelson magnification lens with a field of view of 1.5 × 1.5 mm, Gaussian Regression Filter, a scan speed of 1 × and thresholding of 4	Contour GT-K 3D Optical Microscope (Bruker®), 3D non-contact surface metrology with interferometry	NHT-6	NA
Spectrophotometer	VITA Easyshade® III	Device used to measure wavelength transmitted from one object at a time, without being affected by subjective interferences of color	VITA Zahnfabrik H. Rauter GmbH & Co. KG, Bad Sackingen, Germany	10180	NA
Instant Arabic Qahwa	Baja	Arabic coffee, Cardamom, Cloves, Nondaily Coffee Creamer, Saffron	Baja Food Industrial Co., Saudi Arabia	6281105 795440 Product Date 16/Jan/20120 Expired Date 16/10/2020	NA
Frappuccino cold coffee drink	Starbucks	Semi skimmed milk, Starbucks arabica coffee (water, coffee extract), sugar, natural coffee flavoring, acidity regulator (potassium carbonates), high caffeine content 40 mg/ 100 ml	Arla foods amba dk/8260 viby j. Denmark	5711953024290 Product Date 16/10/2019 Expired on 16/5/2020	NA
Scanning electron microscope	JEOL® JSM-6610 LV Scanning electron microscope	Samples were gold sputter coated with Quorum® Q-150R and after were loaded in the sample stage using 20 kv and 100×, 250× and 500	Akishima, Tokyo, Japan	NA	NA

### Sample preparations and distributions

A total of 96 samples were prepared; 32 samples were CAD/CAM all-ceramic restorative materials i.e., Vita Enamic, Vitablocs Mark II, and Vita Suprinity (VITA Zahnfabrik, Bad Säckingen, Germany). Thirty-two discs of standardized dimensions comprising of each restorative material of controlled/uniform size were milled, consisting of 10 mm × 10 mm ± 0.2 diameters and thickness in the range of 2.0 ± 0.2 mm, following the manufacturer’s instructions using a CAD/CAM machine (Amann Girrbach, Germany) and glazed (Fig. 1).

### Surface treatments of samples

Each group was divided into two equal subgroups of 16 each. One subgroup was obtained as glazed from the laboratory, and further measurements were carried out without any surface treatments. Another subgroup (16 each) was polished with an OptraFine polishing kit (Ivoclar, Vivadent, Germany), as per the manufacturer’s instructions under constant load (2 ± 0.25 kg) using an equal number of grinders in a single direction [27]. Polishing steps are first, finishing = F (light blue), second polishing = P (dark blue). The maximum polishing speed for the used handpiece was 15,000 RPM, which comprised of water spray followed by high Gloss Polishing brush (HP), with a speed of 5000–7000 rpm (Maximum 10,000 rpm) without water and with OptraFine HP Polishing Paste. Sample surfaces were modified to mimic the clinical adjustment of ceramic restorations and polished. A single operator (M.M.) dealt with the specimens according to the instruction presented in the leaflet of each CAD/CAM all-ceramic type.

### Surface roughness measurements

The characterizations and imaging were performed using a Contour GT-K 3D Optical Microscope (Bruker®), a 3D non-contact surface metrology with interferometry. Samples were measured by Vertical Scan Interferometry using a 5 × Michelson magnification lens with a field of view of 1.5 × 1.5 mm, Gaussian Regression Filter, a scan speed of 1x, and thresholding of 4. Samples were placed on the profilometer device and manually adjusted to give an image on the monitor screen. The microscope used Vision 64 (Bruker®) software to control the instrument settings, data analyses, and graphical output. The measurement was performed using vertical scanning interferometry, using broadband (normally white) light source, effective for measuring objects with rough surfaces and objects with adjacent pixel-height differences greater than 135 nm. Each sample was scanned at 3 given points and averaged accordingly to determine the Ra value in micrometer (μm). This was considered as Ra1 value before immersion. Ra measurements followed the ISO 11562 recommendations for standardization [11].

### Color measurements

Color measurements were in the gray background for all the samples using a single operator (M. M.) with the help of Easyshade Vita probe spectrophotometer (VITA Easyshade® III, VITA, Germany). All the samples were measured for the CIE-Lab values to provide the numerical values of the 3D color measurements. L*, a*, and b* values for all the samples were measured thrice, and the average value was considered as ΔE*. The values were presented in the means of the color data and standard deviation (SD) as discussed previously [14, 22–24].

### Sample immersion and thermocycling

Following Ra and color measurements, samples from two subgroups i.e., polished and glazed subgroups from each CAD/CAM all-ceramic restorative material were further divided. Half of the samples were immersed in hot Arabic Qahwa and half in a cold Frappuccino cold coffee drink along with thermocycling.

Instant hot Arabic Qahwa was a form of Nitrogen Flushed Packet for single use. The immersion solution was prepared for each packet (30 g), mixing with 1-L boiled water at 100 C^0^ jack, then boiled for 15 s. Starbucks Frappuccino cold coffee drink is readily available in a sealed bottle, to be shaken well and used. All the samples were immersed, and thermocycling was performed. Fresh solutions were used daily for the samples ready to be immersed. [22, 24, 25] During immersion, the aging process was performed at a temperature between 5 and 55 °C with a total number of 5000 cycles. Following the aging process for 30 days, all the samples were dipped in distilled water 10 times, wiped with tissue paper, and left to dry.

Subsequently, the surface roughness (R2) and color measurements were again carried out with the same instruments and registered as post-aging readings. The mean Ra was calculated before and after immersion (R2-R1). However, mean average color changes (ΔE*) values were calculated using the following equation: ‘’∆E* = [(L1* − L2*)^2^ + (a1* − a2*)^2^ + (b1* − b2*)^2^2]^1/2^ ‘’.(14, 22–25).

### Scanning electron microscope images

A tested glazed or polished sample material and different immersed stain materials were selected for SEM capturing. The samples were gold sputter-coated with Quorum® Q-150R (East Sussex, BN8 6BN, United Kingdom). Then, the samples were loaded into the specimen’s stage of the JEOL® JSM-6610 LV (Akishima, Tokyo, Japan). Samples were scanned and images captured with a Scanning Electron Microscope using 20kv and 250 × magnification.

### Statistical analysis

Mean, SD of Ra, and average ΔE* of glazing and polishing for CAD/CAM all-ceramic restorative samples of VE, VM II, VS were recorded before and after immersion in hot Arabic Qahwa, cold Frappuccino coffee drink, and thermocycling. Microsoft Excel 13 software was used to input the data and analyzed using Statistical Package for Social Science (SPSS) version 22.0 (SPSS Inc., Chicago IL, USA). One-way ANOVA paired Student’s t-test, and Bonferroni test were applied to detect any significant difference between the groups. P > 0.05 was set at the significant level. Then, ΔE* values were linked and compared to reach a clinically acceptable threshold of 4.2 units as mentioned in the previous study. [23] Both ANOVA and Student t-test have been used to compare the values of the color changes or differences between different groups as well as in between intervals time, and also compared color changes of every single group to 50:50% perceptibility and acceptability thresholds to assess if such colour changes are clinically acceptable.

## Results

The mean changes and the Ra SD of the tested ceramic restorative materials were between 0.56 and 0.59 µm. No significant differences were noticed between the tested materials and p-value < 0.940. The highest ΔE^*^ and SD were in the VE group (3.07 ± 0.49), followed by the VM II and VS [(2.65 ± 0.26) and (1.96 ± 0.64)]. Additionally, the ANOVA test for ΔE* exhibited a statistically significant difference between ceramic materials with p-values < 0.001 (Table 2). The Bonferroni post hoc test was conducted for multiple comparisons between the three CAD/CAM restorative materials, which showed significant differences in the ΔE values between the tested CAD/CAM restorative materials with a p-value < 0.001. However, other parameters show no significant differences.

**Table 2:** Mean and standard deviation (SD) of surface roughness (Ra), and mean color change (ΔE*) values of ceramic materials before and after coffee immersion and thermocycling (ANOVA test between and within the groups)

Parameter	Mean ± SD	Ceramic Type	Vita Enamic	Vitablocs Mark II	Vita Suprinity	P value
S Roughness Pre (Ra1)	1.78 (0.55)	Vita Enamic	–	0.436	1.000	0.111
	1.55 (0.46)	Vitablocs Mark II	0.436	–	0.126	
	1.86 (0.76)	Vita Suprinity	1.000	0.126	–	
S Roughness Post (Ra2)	2.37 (0.41)	Vita Enamic	–	0.267	1.000	0.077
	2.11 (0.63)	Vitablocs Mark II	0.267	–	0.095	
	2.44 (0.73)	Vita Suprinity	1.000	0.095	–	
Mean Roughness Changes (Ra)	0.59 (0.40)	Vita Enamic	–	1.000	1.000	0.940
	0.56 (0.53)	Vitablocs Mark II	1.000	–	1.000	
	0.58 (0.38)	Vita Suprinity	1.000	1.000	–	
Mean Color Changes (ΔE)	3.07 (0.49)^a^	Vita Enamic	–	0.002*	0.000*	0.000*
	2.65 (0.26)^b^	Vitablocs Mark II	0.002*	–	0.000*	
	1.96 (0.64)^c^	Vita Suprinity	0.000*	0.000*	–	

Different superscripts upper case letters mean statistical difference inside the respective subgroups (P < 0.001) based on ANOVA followed by Bonferroni tests

**Fig. 2:**
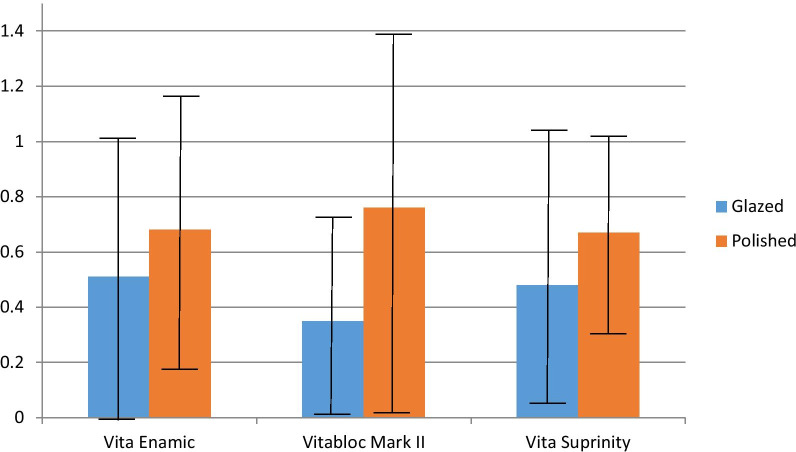
Bar chart representing the mean and SD of Ra values in relation to surface type (t-test)

**Fig. 3:**
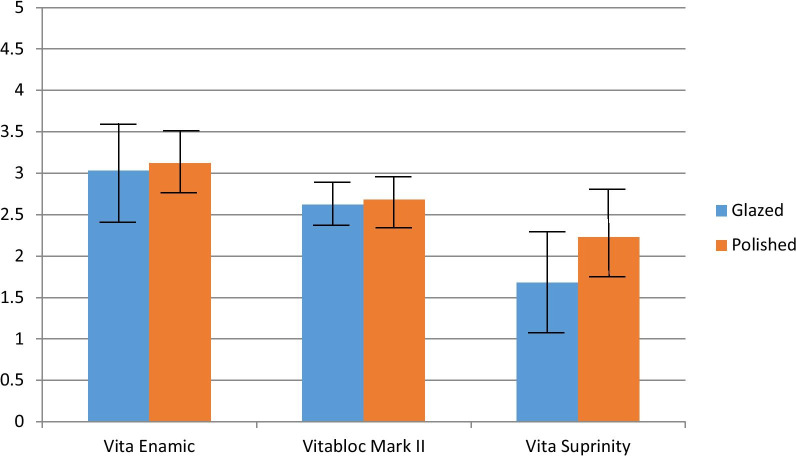
Bar chart representing the mean and SD, and ΔE* values in relation to surface type (t-test)

By comparing surface materials, the Ra means was nearly the same for the three tested types of ceramic and their surface treatment type. The Ra rang was between 0.51 ± **0**.52 µm and 0.35 ± 0.31 µm for the glazed specimens. However, it was in the range of 0.76 ± 0.63 µm and 0.67 ± 0.38 µm for polished specimens of the ceramic materials (Table 3 and Fig. 2). The ΔE* values of glazed and polished VE were the highest ΔE* (3.03 ± **0.59** and 3.12 ± **0.38**), whereas the lowest was for glazed and polished VS (1.68 ± **0.61** and 2.23 ± **0.56**). Student t-test showed a significant difference between glazed and polished VM II in the mean Ra with a p-value < 0.025. However, the ΔE* values are between the glazed and polished VS with a p-value < 0.012 (Table 3 and Fig. 3).

**Table 3:** Mean and standard deviation (SD) of surface roughness (Ra), and color change (ΔE*) values of the ceramic materials after staining immersion and thermocycling in relation to surface treatment type (t-test)

Parameter	Surface treatment type (Mean ± SD)		P value (t-test)
	Glazed	Polished	
*Vita Enamic*			
Surface roughness Pre (Ra1)	1.57 (0.40)	1.98 (0.61)	0.033*
Surface roughness Post (Ra2)	2.08 (0.22)	2.66 (0.22)	0.000*
Mean roughness changes (Ra)	0.51 (0.52)	0.68 (0.49)	0.217
Mean color changes (ΔE*)	3.03 (0.59)	3.12 (0.38)	0.609
*Vitablocs Mark II*			
Surface roughness Pre (Ra1)	1.53 (0.53)	1.75 (0.39)	0.813
Surface roughness Post (Ra2)	1.88 (0.62)	2.34 (0.58)	0.043*
Mean roughness changes (Ra)	0.35 (0.31)	0.76 (0.63)	0.025*
Mean color changes (ΔE*)	2.62 (0.22)	2.68 (0.30)	0.592
*Vita Suprinity*			
Surface roughness Pre (Ra1)	1.38 (0.45)	2.35 (0.70)	0.000*
Surface roughness Post (Ra2)	2.05 (0.48)	2.83 (0.74)	0.001*
Mean roughness changes (Ra)	0.48 (0.37)	0.67 (0.38)	0.157
Mean color changes (ΔE*)	1.68 (0.61)	2.23 (0.56)	0.012*

* = p≤0.05; Significant

The highest Ra means and SD for cold coffee were 1.20 ± 0.57 and 1.00 ± 0.20 for polished VM II and polished VS, respectively. The student t-test is significant among the glazed VE, polished VM Mark II, and polished VS with a p-value < 0.001. The highest ΔE* and SD values for hot Arabic Qahwa were for glazed VE and polished VM II (2.90 ± 0.19 and 2.73 ± 0.23). However, the lowest effects were for glazed VS (1.94 = 0.80). The highest ΔE* for cold coffee was for polished followed by glazed VE with 3.40 = 0.30 and 3.15 = 0.82 respectively. The lowest effects were glazed VS (1.42 = 0.09). The p-values for color changes were significant among the subgroups of polished VE and VS. For multiple comparisons, the one-way ANOVA and Bonferroni post hoc tests showed a significant difference in the mean Ra values among the glazed VE, polished VM II, and VS between immersion types with p-values < 0.016, 0.002, and 0.000, respectively. A significant difference in ΔE* was noted in the polished subgroup of VE and VS in both immersion materials compared with P-values < 0.001 and 0.003 (Table 4).

**Table 4:** Mean and standard deviation (SD) for different types of ceramic with different surfaces treatment types and immersion type (t-test)

Ceramic and surface treatment types	Immersion type	Surface roughness Pre (Ra1)	Surface roughness Post (Ra2)	Mean roughness changes (Ra)	Mean color changes (ΔE*)
Vita Enamic Glazed	Arabic Qahwa	2.39 (0.33)	2.80 (0.16)	0.40 (0.35)	2.90 (0.192)
	Cold Coffee	1.57 (0.55)	2.53 (0.19)	0.96 (0.46)	3.15 (0.82)
	P value (t-test)	0.003*	0.008*	0.016*	0.436
Vita Enamic Polished	Arabic Qahwa	1.67 (0.48)	2.18 (0.32)	0.51 (0.31)	2.84 (0.20)
	Cold Coffee	1.47 (0.31)	1.98 (0.37)	0.50 (0.20)	3.40 (0.30)
	P value (t-test)	0.336	0.244	0.955	0.001*
Vitablocs Mark II Glazed	Arabic Qahwa	1.38 (0.32)	1.76 (0.39)	0.38 (0.29)	2.67 (0.20)
	Cold Coffee	1.69 (0.68)	2.01 (0.80)	0.33 (0.33)	2.58 (0.24)
	P value (t-test)	0.271	0.430	0.768	0.418
Vitablocs Mark II Polished	Arabic Qahwa	1.81 (0.36)	2.13 (0.61)	0.32 (0.28)	2.73 (0.23)
	Cold Coffee	1.33 (0.26)	2.54 (0.50)	1.20 (0.57)	2.62 (0.37)
	P value (t-test)	0.010*	0.165	0.002*	0.471
Vita Suprinity Glazed	Arabic Qahwa	2.63 (0.65)	3.30 (0.56)	0.66 (0.42)	1.94 (0.80)
	Cold Coffee	2.07 (0.68)	2.37 (0.60)	0.30 (0.22)	1.42 (0.09)
	P value (t-test)	0.108	0.007*	0.053	0.111
Vita Suprinity Polished	Arabic Qahwa	1.55 (0.42)	1.90 (0.51)	0.35 (0.19)	2.61 (0.22)
	Cold Coffee	1.21 (0.44)	2.20 (0.43)	1.00 (0.20)	1.86 (0.55)
	P value (t-test)	0.129	0.217	0.000*	0.003*

* = p≤0.05; Significant

The generalized linear model between individual variations and the two-way repeated measurements using ANOVA showed a significant difference for each surface treatment, immersion type interaction between ceramic type and surface treatment protocol as well as immersion type (P ≤ 0.05) between the three parameters. However, no significant differences were found regarding the interaction between ceramic (Ra) type and immersion type (ΔE*) (Table 5).

**Table 5:** Two-way repeated measure ANOVA of ceramic materials, immersions, and surface roughness types (Account for within individual variations and between individual variations)

Parameter	Changes in surface roughness (Ra)			Mean color changes (ΔE*)		
	Type III Wald Chi-Square	Df	Sig	Type III Wald Chi-Square	Df	Sig
Type of ceramic	0.240	2	0.887	131.447	2	0.000*
Surface treatment	4.967	1	0.026*	8.268	1	0.004*
Immersion type	18.899	1	0.000*	1.944	1	0.163
Type of ceramic * Surface treatment	14.004	2	0.001*	8.014	2	0.018*
Type of ceramic * Immersion type	2.868	2	0.238	27.698	2	0.000*
Surface treatment * Immersion type	12.435	1	0.000*	0.010	1	0.919
Type of ceramic * Surface treatment * Immersion type	31.471	2	0.000*	2.004	2	0.367

* = p≤0.05; Significant

**Fig. 4:**
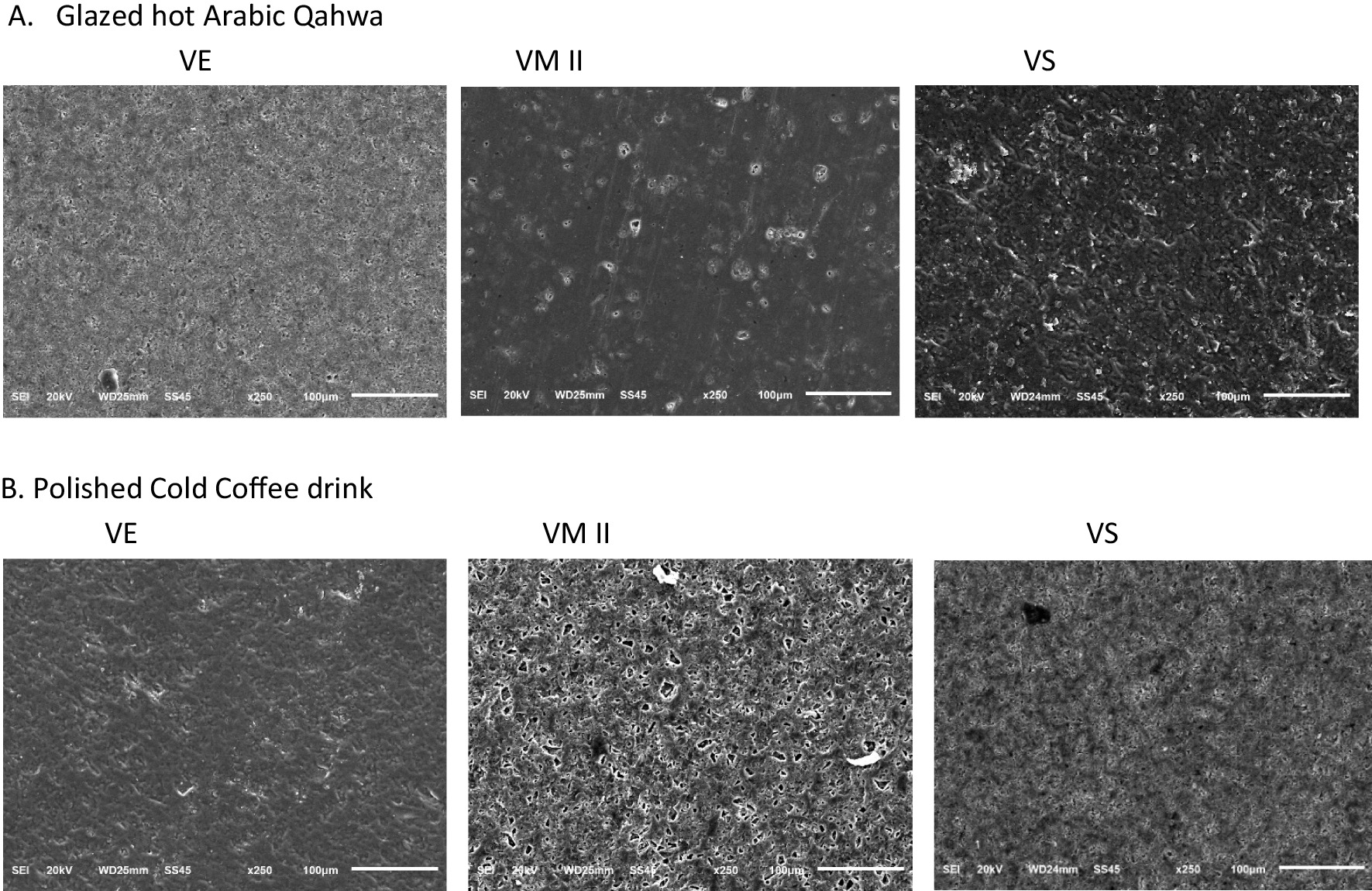
SEM images of tested CAD/CAM restorative materials ‘’VE, VM II, VS’’ at X 250 magnification after immersion in staining materials and thermocycling. **A** Glazed hot Arabic Qahwa, **B** Polished Cold Coffee sample

Figure 4 shows the SEM images for all the glazed and polished CAD/CAM materials after immersion and thermocycling. Polished samples have more flaws compared to the glazed ones. In Glazed Hot Arabic Qahwa, the image VM II sample has a smoother surface compared to other ceramic materials. While VE has a smoother surface for polished Cold coffee samples confirming the profilometry measurements.

## Discussion

Patients are increasingly demanding anterior aesthetic restorations owing to their long-term color stability and continuous development for All-ceramic materials. Following consumption of hot and cold beverages, some degree of color changes is noticed. Different types of Arabian Qahwa coffees contain Saffron, Ginger, Cardamom, natural coffee flavoring, potassium carbonates, and high caffeine content, which might be a staining factor to the intraoral cemented aesthetic prosthesis [22, 26]. This in-vitro spectrophotometric study evaluated the effect of surface roughness and color changes on different CAD/CAM restorative ceramic materials following immersion in hot Arabic Qahwa and Frappuccino cold coffee drinks separately. The null hypothesis was accepted since no significant difference in Ra is noticed in correlation to the different groups. However, a significant difference in ΔE* of tested materials was detected. A significant variance is noticed in mean Ra between glazed VE and polished surfaces of VM II and VS and between polished VE and VS subgroups in ΔE* of coffee type.

### Surface roughness and mean color change

The Ra mean values for the tested CAD/CAM restorative materials were 0.59 μm (VE), 0.56 μm (VM II), and 0.58 μm (VS), higher than the value recorded by Egilmez et al.[3] (0.012) for VE. However, Ra mean was lower than the values obtained by Al Moaleem et al. [14] (1.26) for VM II, Vasiliu et al. [28] (0.12) for feldspathic porcelain, and Qabel et al.[29] (0.17) for CAD/CAM materials. Although the recorded ΔE* values were 1.96 (VS), 2.65 (VM II), and 3.07(VE) within the clinically acceptable values for the three tested materials, these values are consistent with Subaşi et al. [30] (slightly above 2) for VS, Sarikaya et al.[18] (2.094) for VM II, and Colombo et al.[31] (< 3.3) for Zirconia. However, ΔE* values were lower than (3.83 and 4.9) recorded by Saba et al. [17] for VE and VM II and Abu-Obaid et al.[32] (< 3.3 for VE, VM II, VS). However, the values were higher than that of Alp et al.[12] and Al Moaleem et al. [14] who documented lower values than 1.0 for VS and 0.73 for VM II and Zirconia.

Surface roughness plays a crucial role in maintaining the surface values and preventing extrinsic discoloration [11]. Results showed that the surface roughness of the samples before and after thermocycling differed and that thermocycling had a significant impact on Ra, especially on the polished surfaces. The mean Ra values were 0.51, 0.35, 0.48 and 0.68, 0.76, 0.67 μm for glazed and polished samples of VE, VM II, and VS, respectively. Similar values were obtained by Alhabdan et al.[33] who recorded 0.56 μm for glazed surfaces of feldspathic ceramic. However, these were higher than values recorded by Flury et al.[34] 0.20 μm for VM II after polishing, much lower than glazed and polished values recorded by Kozmacs et al.[11] for monolithic zirconium dioxide crowns.

### Surface treatments and Ra measurements

Milled zirconia that reinforced lithium disilicate glass‐ceramic (VS) showed more significant changes when compared to the heat‐pressed glossing and Ra. For VS before and after thermocycling, Vasiliu et al.[28] reported Ra values of 0.02 and 0.04 μm for glazed samples. However, Ra values were 0.3 μm for polished samples. Similarly, VS exhibited significantly lower roughness (0.69 µm) and higher gloss when compared with E.max CAD/CAM restorative material [35]. In another study, the Ra values recorded in VM II before and after thermocycling were 0.1 and 0.08 μm for glazed samples, whereas they were 0.25 and 0.26 μm for polished samples [28]. The present study records higher Ra values for glazed and polished samples for VS (0.48 and 0.67 μm) and VM II (0.35 and 0.76 μm), which may be attributed to the different polishing techniques, materials, and the Ra machine used. Egilmez et al.[13] stated that the tested materials exhibited different Ra values and surface topographies for VE (0.011 µm), lower than values of 0.51 and 0.68 μm for glazed or polished tested samples of the present study. The difference may be attributed to the technique used for sample preparation or the machine for Ra measurement. Al Moaleem et al.[14] showed mean Ra for glazed and polished Vitablocs Mark II (1.26 and 1.93 μm), higher than the mean values obtained in the current study (0.35 and 0.76 μm, respectively). The same study recorded Ra of 1.32 and 2.23 μm for glazed and polished Zirconia samples showing significant differences between VM II and Zirconia. The results of the present study are the same for the glazed and polished samples of both VM II and VS, the sign between the two subgroups.

Glazing or manual finishing and polishing are the most effective procedures that yield higher gloss and minimize the roughness of CAD/CAM silica-based VS. Overall, VS exhibited higher polishability than IPS E.max CAD [35], consistent with the results of the present study, providing slightly similar values for polished or glazed samples.

### Surface treatments and mean ΔE* measurements

The external intraoral prostheses can be glazed or polished surfaces. The glaze layer is significant to the color stability of all-ceramic prostheses. The unglazed surfaces after occlusal intraorally adjustments should be avoided, and careful occlusal evaluation before cementation should be done [36]. The highest mean ΔE* was 3.03 for glazed VE and 3.12 for polished VE samples (without significant differences). Those values were slightly higher but somewhat lower than the values obtained (3.17–3.56) for VE polished samples [16], and this range was clinically acceptable. Abuobaid et al. [32] recorded ΔE* 2.54 ± 0.27 for polished VE and 1.17 ± 0.59 for reglazed samples. Vasiliu et al.[28] documented 1.35 for the hybrid ceramic, after being exposed to hot and cold coffee. Acar et al. [21] concluded that VE glazed specimens were lower ΔE* than polished specimens for all-ceramic materials tested. In addition, when polished with different polishing materials, ΔE* VE values were at an acceptable level for all the ceramic materials. All these values [21, 28, 32] are slightly lower than the VE values of the present study despite that all ΔE* are clinical in acceptable range (1 < (ΔE*—< 3.7) [18, 22].

For feldspathic CAD/CAM restorative materials (VM II), Sarikaya et al. [18] proposed that the feldspathic and low-fusing porcelain specimens were more color-stable as glazed versus polished ones regardless of whether stained with the coffee solution or other staining materials. VM II in form of glazed and polished samples with different polishing materials demonstrated that the ΔE* values were at an acceptable level (1 < (ΔE*—< 3.7) without significant differences between all-ceramic tested materials [18]. In the present study, no significant differences were found between glazed (2.62) and polished (2.68) based on clinically acceptable color changes. Abu-Obaid et al. [32] recorded 2.39 for polished VM II samples after immersing in coffee. All the tested glazed or polished feldspathic metal-ceramic materials shown significant differences in the parameter of ΔE* for the tested groups after immersing in Khat extract [37].

Concerning ΔE* of VS, significant differences were present between the glazed (1.68) and polished (2.23) samples, although these values were slightly lower than values recorded 2.36 ± 0.66 for polished samples after staining and 0.99 ± 0.54 after re-glazing [32]. Polished surfaces after crown recontouring or occlusal adjustments should be avoided and reglazed before definitive cementation [22, 24]. Alp et al. [12] concluded that material type had a significant effect on the color difference after coffee immersion and thermocycling. However, the values were within the clinical acceptability threshold (< 1.8 units). Similarly, a significant difference was detected between glazed and polished vs. subgroups samples with a p-value < of 0.012 in the existing study. Additionally, significant color changes after immersion and thermocycling in acidic or Arabic coffee drinks were noticed. However, these changes were more considerable in polished porcelain specimens than those in glazed specimens for all CAD/CAM restorative materials [22, 24].

### Thermocycling and measurements

Thermocycling is a widespread process of artificially enhanced ceramics aging since it replicates the oral environment as an extrinsic influence [8, 30, 38]. The water aging procedure contains identical thermal variations with baths extending from − 5 to 55 °C for several cycles. This process can affect the durability of the prosthesis, and it can simulate the performance of the ceramic restoration in the oral environment [8, 39]. Thermocycling with coffee immersion produced a significant mean ΔE* among monolithic teeth, base acrylic resin, and conventionally processed acrylic resin materials, used for dentures fabrications when compared with red wine [39]. The aging processes for glazed or polished samples of ceramic affected milled ceramic more than the heat press. In addition, the Zirconia reinforced lithium silicate glass‐ceramic experienced a more significant change when carrying out color parameter values [28]. This was consistent with the results of the current study since VE recorded the highest ΔE* among glazed 3.15 and polished samples 3.40 of the cold coffee drink when compared with other tested materials after coffee staining and immersion. Acar et al. demonstrated that thermocycling in hot or cold coffee caused a clinically unacceptable color change for resin nano-ceramic and nano-composite resin materials as compared with hybrid ceramic VE, which was clinically acceptable and lower than 2.0 unit [21]. In this study, glazed samples of VE demonstrated less color change than polished specimens for all-porcelain materials tested after hot and cold coffee immersion and thermocycling.

Abu-Obaid et al. [32] estimated ΔE* values for the glazed VE ceramic materials after hot coffee staining (2.54 ± 0.27). Vasiliu et al.[28] concluded that ΔE* of VE or hybrid ceramic was 1.35 after being exposed to hot and cold coffee, and the outcome was clinically acceptable for the tested thickness. Sagsoz et al. [40] recorded ΔE* of 2.0 and less for glazed VE after 4 weeks of coffee staining. In contrast to the results obtained by El Sayed et al. [41] who recorded a high and unacceptable clinical average color change (7.95 ± 0.36) for the VE ceramic and ΔE* of 4.90 for VE after coffee immersion. [17]

Sarikaya et al. [18] suggested that VM II specimens were more color-stable for glazed than polished, regardless of whether stained with the hot coffee solution or other cold staining materials. Palla et al. [36] stated that Vita Mark II in form of glazed and polished samples using different polishing materials were acceptable ΔE* for all tested ceramic materials. Another study recorded the ΔE* (2.39 ± 0.54) for VM II upon staining in hot coffee after 4 weeks [32]. All previously mentioned values are similar to the results obtained in this study, which are between 2.67 and 2.58 for glazed samples and 2.73 and 2.62 for polished samples immersed in Arabic Qahwa and cold coffee, respectively. A higher value is registered for glazed samples of VM II (3.83) after coffee immersion [17]. To raise the discoloration resistance, glazing is recommended after surface alteration for all the CAD/CAM restorative materials [32].

After exploring the effect of coffee staining solution with 5000 thermocycling cycles for glazed Zirconia reinforced lithium, silicate VS samples were significant (ΔE*) when compared with other tested materials, which were within the clinically acceptable parameters [30]. This study showed significant differences (ΔE*) among polished specimens and within acceptable limits after hot or cold coffee immersions and thermocycling. Vasiliu et al.[28] showed that VS color and feldspathic glass‐ceramic were more affected by the aging and thermocycling, causing glazed samples to be rougher, having a significant impact on color translucency (lesser than 2 for VS samples). Alp et al. [12] concluded that VS and lithium disilicate treated with different surface finishing procedures (glazing or polishing) have significant differences and clinically acceptable for color changes after coffee thermocycling. This is consistent with the findings of this study, showing significant differences of P-value < 0.003 between glazed and polished samples after hot or cold coffee immersion with the VS samples.

Significant differences for Ra were shown between the different tested parameter (ceramic, surface treatment, and immersion), consistent with previously published results [13, 28, 35]. The same findings were recorded in the ΔE*values after immersion and color change measurements, consistent with previous studies [12, 13, 32, 36, 38] that demonstrated significant differences between two surface treatments and material types. This study was conducted with only one hot, cold coffee, and polishing with one selected kit. Future studies should focus on other staining solutions, polishing kits, and more samples.

## Conclusions

This colorimetric in-vitro study showed that after surface treatments and staining with different coffee solutions:

- The mean Surface roughness increased for all sample groups and also the mean colour changes with the highest differences in VE and those in VS but within clinically acceptable limits.
- Increased surface roughness was observed in all the glazed with polished subgroups post-immersion and thermocycling. It was highest for polished VM II group. However, mean color changes higher values were noticed for VS group but within range of clinical acceptance.
- Within glazed immersion groups, no mean color changes were observed however, surface roughness changes were higher in VE cold coffee samples. Within polished immersion groups, higher Ra changes were found in VM II and VS cold coffee subgroups. Substantial mean color changes were also recorded in VE (cold coffee) and VS (Arabic Qahwa) polished subgroups but within clinical acceptance.
- SEM images showed more roughness for the polished samples than the glazed samples.

## Data Availability

The datasets used and/or analyzed during the current study available from the corresponding author on reasonable request.

## Abbreviations

Ra: Surface roughness
CAG/CAM: Computer-aided design/computer-aided manufacturing
ΔE: Mean color changes
VS: Vita Suprinity
VE: Vita Enamic
VM II: Vitablocs Mark II
